# Increased transmission of SARS-CoV-2 in Denmark during UEFA European championships

**DOI:** 10.1017/S095026882200019X

**Published:** 2022-03-23

**Authors:** Marc Bennedbæk, Mia Sarah Fischer Button, Lise Birk Nielsen, Jonas Bybjerg-Grauholm, Christina Wiid Svarrer, Karina Lauenborg Møller, Brian Kristensen, Rebecca Legarth, Vithiagaran Gunalan, Ditte Rechter Zenas, Irfatha Irshad, Sophie Gubbels, Raphael N. Sieber, Marc Stegger, Palle Valentiner-Branth, Morten Rasmussen, Camilla Holten Møller, Jannik Fonager, Frederik Trier Moller

**Affiliations:** 1 Virus and Microbiological Special Diagnostics, Statens Serum Institut, Copenhagen, Denmark; 2 Division of Infectious Disease Preparedness, Statens Serum Institut, Copenhagen, Denmark; 3 Department for Congenital Disorders, Statens Serum Institut, Copenhagen, Denmark; 4 Department of Bacteria, Parasites and Fungi, Statens Serum Institut, Copenhagen, Denmark

**Keywords:** Epidemiology, SARS-CoV-2, transmission, mass events, public health

## Abstract

Denmark hosted four games during the 2020 UEFA European championships (EC2020). After declining positive SARS-CoV-2 test rates in Denmark, a rise occurred during and after the tournament, concomitant with the replacement of the dominant Alpha lineage (B.1.1.7) by the Delta lineage (B.1.617.2), increasing vaccination rates and cessation of several restrictions. A cohort study including 33 227 cases was conducted from 30 May to 25 July 2021, 14 days before and after the EC2020. Included was a nested cohort with event information from big-screen events and matches at the Danish national stadium, Parken (DNSP) in Copenhagen, held from 12 June to 28 June 2021. Information from whole-genome sequencing, contact tracing and Danish registries was collected. Case–case connections were used to establish transmission trees. Cases infected on match days were compared to cases not infected on match days as a reference. The crude incidence rate ratio (IRR) of transmissions was 1.55, corresponding to 584 (1.76%) cases attributable to EC2020 celebrations. The IRR adjusted for covariates was lower (IRR 1.41) but still significant, and also pointed to a reduced number of transmissions from fully vaccinated cases (IRR 0.59). These data support the hypothesis that the EC2020 celebrations contributed to the rise of cases in Denmark in the early summer of 2021.

## Introduction

On 30 January 2020, the coronavirus disease (COVID-19) outbreak was declared a Public Health Emergency of International Concern (PHEIC), and on 11 March 2020, the WHO declared the COVID-19 outbreak a pandemic. The first case was confirmed in Denmark on 27 February 2020.

Denmark hosted four games in the 2020 UEFA European championships (EC2020), with the tournament lasting from 11 June to 11 July 2021. After the initial group stages hosted in Denmark, the team reached the semifinal, with widespread celebrations ensuing. The events took place following a period of declining rates of infections in Denmark, and cessation/lifting of several restrictions, increasing Delta (B.1.617.2) transmission and increasing vaccination coverage from 25.1% on 12 June 2021 to 39.8% on 12 July 2021, mainly among persons born in 1957 or later [1]. Access to many locations, including the events at the Danish national stadium, Parken (DNSP), and big-screen events, was conditional on proof of completed vaccination, previous COVID-19 infection or a negative test less than 72 h old, resulting in a valid Digital COVID-19 Certificate. At the time of the study period, a large proportion of the Danish population of 5 843 347 was tested with PCR or antigen test on a weekly basis with over ~500 000 and ~1 000 000 tests per week, respectively (https://www.dst.dk/da/Statistik/emner/borgere/befolkning/befolkningstal (2021) (Accessed 29 November 2021)). In addition, a massive effort to trace contacts of COVID-19 infected was in place, with more than 95% of cases being in contact with contact tracing staff from the Danish Patient Safety Authority [1] (https://stps.dk/da/sundhedsfare-og-beredskab/coronaopsporing/data-om-smitteopsporing-af-smittede/). Finally, more than 90% of SARS-CoV-2-positive samples underwent whole genome sequencing (WGS) in Denmark, which allowed for identification of possible transmissions [2].

It is widely accepted that large gatherings of people increase the risk of transmission of SARS-CoV-2 and that events with large crowds can result in a high number of transmissions [3–5]. Authorities in Denmark decided to implement enhanced monitoring of COVID-19 spread during the EC2020 celebrations. Information on the total number of spectators at a series of big-screen events, with public broadcasting of the matches and main game events at the DNSP, was collected. In addition, already established surveillance data from cases who participated in such venues were retrieved from the Danish Patient Safety Authority.

We hypothesise that EC2020 matches, gatherings and celebrations contributed to the Danish COVID-19 wave in the early summer of 2021. The hypothesis is based on the observed rise in COVID-19 cases, concomitant with the games, and previous studies supporting that singing, alcohol consumption and large gatherings may lead to increased risk of SARS-CoV-2 transmission [6, 7]. In this study, we conducted a population-based study to estimate whether EC2020 celebrations resulted in increased SARS-CoV-2 transmissions through analysis of likely transmission pairs, derived reproductive numbers, number of contacts, among all cases that were infectious or not infectious on match days. Further, we aim to assess the possible transmission of SARS-CoV-2 at selected organised public venues requiring valid Digital COVID Certificates in Denmark and compare this to population-based estimates from the same period to evaluate the effect of measures taken to reduce the spread of COVID-19 during the EC2020.

## Methods

### Population

The study included all persons with a positive test for SARS-CoV-2 in Denmark from 30 May 2021, 14 days before the EC2020 until 25 July 2021, 14 days after the EC2020. Attendance information traced by the Danish Patient Safety Authority, and any cases linked to any of the big-screen events held on each match day during EC2020, and the four main game events at the national stadium in Denmark, was included in this nested cohort study.

### Data sources

In Denmark, the Danish Civil Registration System containing information on vital status and previous and current addresses enables linkage between a multitude of registries through a Personal Identification Number (PIN) [8].

SARS-CoV-2-positive tests during the period of study were identified using the Danish Microbiology Database (MiBa) that includes information on all SARS-CoV-2 samples and their results for all individuals tested for SARS-CoV-2 by RT-PCR and antigen tests in Denmark [9, 10], the latter of which have been in use since December 2020. In the case of a positive antigen test result, only persons with confirmatory RT-PCR tests were included in the study. The timeframe was too short for any reinfections within 90 days to occur; thus, a person could only have one SARS-CoV-2 infection during this period.

Information on cases was expanded using data from other national registries, including the automated COVID-19 surveillance system at Statens Serum Institut (SSI; Copenhagen, Denmark), which is described in detail elsewhere [11]. The COVID-19 surveillance system uses the PIN to collect information from the National Patient Registry, and other registries including the Danish Vaccination Registry DDV [12]. The population reproduction number was calculated by the Danish mathematical modelling expert group that has been modelling the pandemic in Denmark since the onset of the pandemic (https://covid19.ssi.dk/analyser-og-prognoser/modelberegninger/ekspertgruppen-for-matematiske-modelberegninger (2021) (Accessed 29 September 2021)). Information on addresses allowed for the identification of shared epidemiological links such as shared households and staircases, necessary to identify possible transmissions between cases. Data from the National Agency for IT and Learning allowed for the identification of schools, and hence identification of transmissions likely to have occurred between SARS-CoV-2-positive cases attending the same schools (https://www.stil.dk/ (Accessed 25 August 2021)).

WGS was performed by the Danish COVID-19 Genome Consortium (DCGC) on ~90% of all positive samples available for sequencing in Denmark, resulting in 70–80% usable genomes, and was a coordinated effort by SSI and regional hospitals across Denmark (https://www.stil.dk/ (Accessed 25 August 2021)).

Finally, the Danish Patient Safety Authority provided data from contact tracing activities, including whether cases were linked to specific outbreaks or selected venues mentioned above, including the date and location of outbreaks. The Danish Patient Safety Authority is in contact with more than 95% of all SARS-CoV-2 infected in Denmark for contact tracing. For this analysis, only cases linked to outbreaks with more than five cases were included. In addition, contact tracing of tourists is likely to be less complete (https://stps.dk/da/sundhedsfare-og-beredskab/coronaopsporing/data-om-smitteopsporing-af-smittede/). The Danish Football Association provided attendance statistics for all matches and selected venues (https://www.dbu.dk/).

### Study design and definitions

Transmission events between a primary case and a secondary case were regarded as possible if there was an identifiable epidemiological linkage between cases. This linkage could be from registries, i.e. shared household, staircase or school or a link identified through contact tracing, when both cases were identified to be part of the same outbreak defined by the Danish Patient Safety Authority. Further, a shared time window was required, as the time from the onset of infectiousness between one case and onset of symptoms or sample date of the suspected linked case was restricted to 14 days.

For the 28 325 cases with high or medium quality genomes (defined as having an n-count<3000), a simple crude genetic distance, in terms of nucleotide (nt) substitutions, nt deletions, between case–case pairs, could be calculated. All possible transmissions with a pairwise genetic distance above 3.5 were excluded (Supplementary Fig. S1). The pairwise genetic distance cut-off was based on the inclination of the curve (Supplementary Fig. S2); see results section on phylogenetic analysis. For completeness, cases with missing sequences or lower quality genomes were also included in the analysis and assigned an arbitrary genetic distance between pairs of 3.5, to avoid excluding pairs exclusion with missing sequence information.

**Fig. 1.**
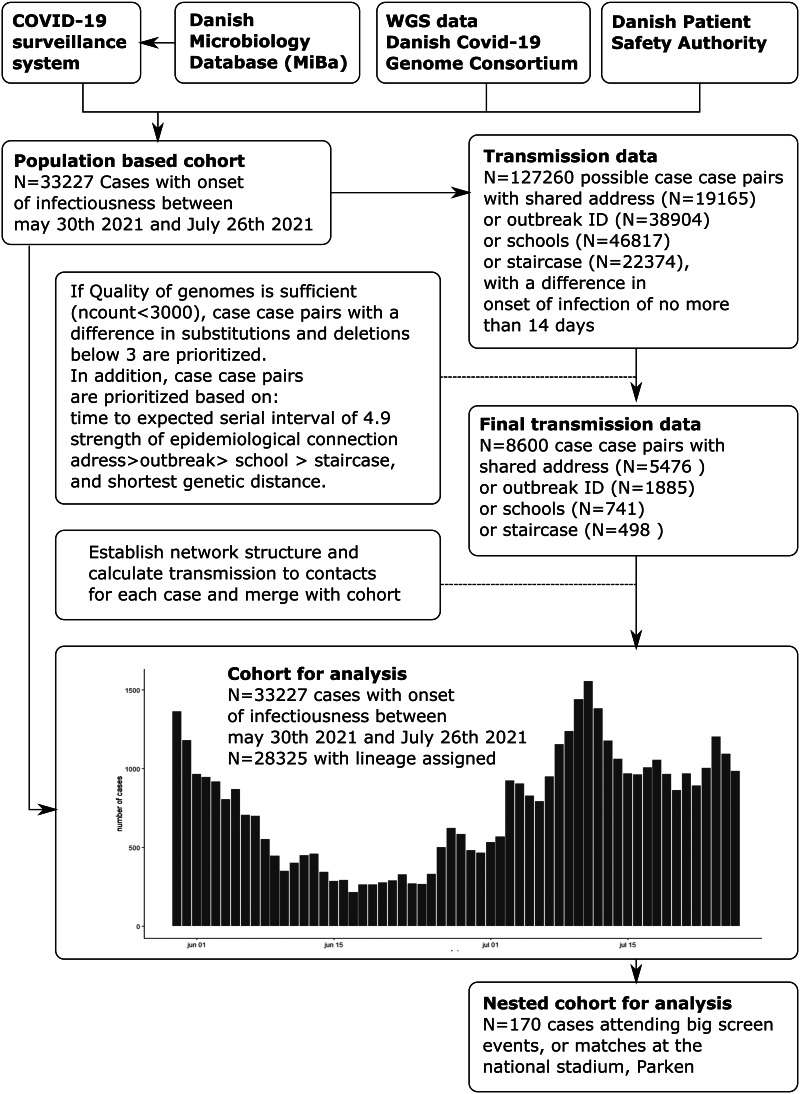
Flowchart of the included cases and the creation of possible transmission events, transmission trees and analysis dataset, and epicurve of the time period.

In outbreaks with multiple infected that are epidemiologically linked, finding the most likely transmission route or probable transmission source for each case might prove challenging, and even more so in larger outbreaks. Many different approaches to this have been proposed and have been evaluated to be more efficient than phylogenetic analysis alone [13–15]. To establish a probable source of transmission to each secondary case, we first selected transmissions, closest to the expected average serial interval between the expected onset of disease of the two cases. The expected average serial interval was set to 4.9 days, and was based on results from a recent meta-analysis [16]. We then selected the epidemiological connection that was most likely in the following order (household transmissions, outbreak transmissions, school and staircase transmissions). Finally, if the two above criteria were tied, the shortest genetic distance was chosen, where this information was available. The flow of data is depicted in Figure 1. As a result, a primary case could have multiple secondary cases whereas a secondary case could only have one primary case, or no known primary case.

For each SARS-CoV-2 case, we calculated the number of days from onset of infectiousness to test as spanning 2 days before symptom onset, or sample date, whichever came first, until the sample date. For the purpose of adjustment to applied regression analysis, cases were regarded as fully vaccinated 14 days after the second vaccination.

To access the effect of EC2020 celebrations, we defined cases as ‘infectious on match days’ if the timeframe from onset of infectiousness to test covered any of the days the Danish teams played. Thus cases that were not infectious until after the event were defined as cases infected at the event/match day if epidemiologically linked to case infectious at the match days. Cases that were not infectious on match days, i.e. having an onset of the infectious period after the match days or test date before the match day, were defined as not infectious at match days. Other cases were defined by whether they had participated at the DNSP or big-screen events (Fig. 1).

### WGS and phylogenetic analysis

When a positive sample was identified by using RT-PCR, a new aliquot of the SWAP eluate was transferred to a positive plate. RNA was extracted using RNAvanced (Beckman Coulter, Pasadena, CA, USA). According to the manufacturer's instructions, sequencing libraries were then prepared using all reagents included in COVIDSeq Test RUO (Illumina, San Diego, CA, USA). Briefly, in batches of 384 samples, RNA samples were subjected to PCR amplification in two reactions according to the ARTIC version 3 scheme [17]. Then combined before library prep using the DNA prep module, libraries were indexed using a unique dual index, before being bead normalised and pooled. The pool was normalised and sequenced using 74 bp long paired-end reads.

For phylogenetic analysis, sequences from cases with records of attending one or more of the four matches at the DNSP or at big-screen events were retrieved. All sequences had a lineage assigned using the Pangolin tool (version 3.1.11). Sequences were aligned using MAFFT version 7.487 using the command: mafft-linsi <input_sequences.fasta> <output_sequences.fasta>.

Phylogenetic inferences were made for all four matches and big-screen events. The phylogenetic trees were made using IQTree version 2.1.2, and the best-suited substitution model was found using modelfinder in IQ-Tree. The substitution model used was a generalised time reversible model transition model with empirical base frequencies and invariant sites. The tree reliability was estimated using ultrafast bootstrapping with 1000 replicates. The phylogenetic trees were made using the following command: iqtree2 –s <input_sequences_aligned.fasta> -m GTR + F + I —ufboot 1000.

The pairwise cophenetic distance between cases attending the four matches at DNSP or at big-screen events was calculated using the ape package in R version 4.1.1 (2021-08-10) in order to allow for later comparison with the simple pairwise differences in the number of substitutions and deletions.

### Statistical analysis

R version 4.1.1 (2021-08-10) was used to conduct the statistical analysis. For most analyses, simple descriptive statistics were employed. For all cases, whether they were infectious or not infectious on a match day, the number of cases where a plausible link was found, within the next 7 days, was calculated. For the games at the DNSP, a 7-day incidence was calculated directly from the number of cases with a known transmission link for the analysis of the events at DNSP. Thus this incidence was calculated based on cases that could be linked to the previous cases, and results are thus expected to be smaller than the true incidence, had we been able to assess all possible links between cases. For comparison, the national 7-day incidence per 100 000 was calculated using the size of the Danish population as the denominator. To analyse transmission between cases, the epicontacts package version 1.2.0 was used [18]. Briefly, the package allows for the construction of transmission trees of all identified case–case pairs, using the possible pairwise transmission links as described above. The number of transmissions, including the number of transmissions from each case, was derived. For the analysis of differences in the number of transmissions linked to each case, a negative binomial model was selected using the MASS package version 7.3-54. The response variable was the number of transmissions from each primary case, as characterised by the exposure variables. The model was checked using the dispersion parameter and by comparing the observed number of zeros with the expected number.

## Results

### Population-based analysis

A cohort of all known SARS-CoV-2 positives (*N* = 33 227) was identified from 30 May 2021, 14 days before the EC2020 started until 25 July 2021, 14 days after the EC2020 ended (Fig. 1). A total of 8600 unique case–case transmissions were found, which translates to 25.8% of all cases. The 7-day incidence per 100 000 was found to reach a maximum of 119 on 10 July 2021, and a minimum of 21.64 on 19 June 2021, with a median of 64.5 and a mean of 66.26.

**Fig. 2.**
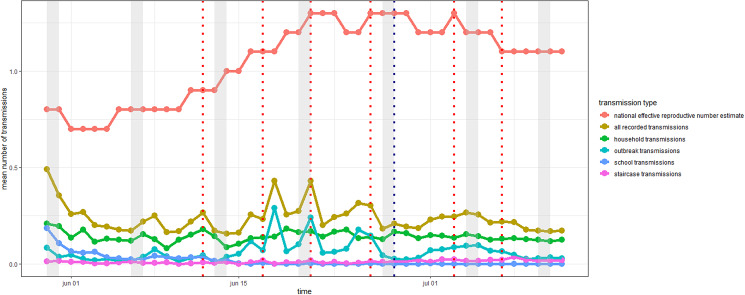
Time series of the national effective reproductive number estimate and the mean number of transmissions to cases by type of transmission. The type of transmission is the epidemiological links defined between cases. These are shared households, living in on the same address, and staircase where the address is the same, but different floor/side. Transmission types also include case–case pairs attending the same school or being registered in the same outbreak. Dotted vertical lines in red depict days were the Danish national team played. The dotted blue line depicts the date of the round of 16 matches between Croatia and Spain at the DNSP, Denmark. Grey bars represent weekends.

The average number of transmission to other cases from each case (out-nodes) by the type of transmission in addition to the population reproductive number for the timeframe is shown in Figure 2. The highest rate of transmission found was 1.88 from the 44 people infectious on match days, who attended the matches at DNSP or big-screen events (Table 1).

**Table 1.** Transmissions, contacts and incidence by infectiousnes at match days

	Cases not infectious at match days	Select cases attending national stadium and events	Total cases infectious on any match day	Cases infectious at single match days					
				12 June	17 June	21 June	26 June	3 July	7 July
*N* infectious cases	26 122	44	7105	949	626	675	884	1574	2397
*N* days from onset of infectiousness to test	80 996	135	24 079	3338	1856	2101	3122	4541	9121
*N* close contacts to cases	88 121	593	41 478	4366	3558	5398	9775	8484	9897
*N* transmissions to secondary cases	6044	83	2556	314	178	317	413	485	849
*N* transmissions to household secondary cases	4058	15	1418	196	95	160	198	286	483
*N* transmissions to outbreak secondary cases	942	68	943	65	70	151	199	157	301
*N* transmissions to school secondary cases	681	0	60	48	9	1	1	1	0
*N* transmissions to staircase secondary cases	363	0	135	5	4	5	15	41	65
Rate of transmission from cases^a^	0.23	1.88	0.36	0.33	0.28	0.47	0.47	0.31	0.35
Attack rate^b^	0.07	0.06	0.06	0.07	0.05	0.06	0.04	0.06	0.09
*N* contacts to each case	3.37	13.47	5.84	4.60	5.68	8.00	11.06	5.39	4.13
Rate of transmissions by each infectious day^c^	0.07	0.61	0.11	0.09	0.10	0.15	0.13	0.11	0.09

a Rate of transmission from cases was calculated as number of identified *N* transmissions to secondary cases divided by *N* infectious cases.

b The attack rate was calculated as the number of identified *N* transmissions to secondary cases divided by *N* close contacts to cases.

c Rate of transmissions by each infectious day cases was calculated as the number of identified *N* transmissions to secondary cases divided *N* days cases was infectious.

The increased number of transmissions to secondary cases and higher number of close contacts when comparing cases infectious at match days to cases not infectious at match days are shown in Table 1. The number of close contacts to each case was 5.84 on match days compared to other days (3.37), whereas the attack rate was only 0.01 higher for cases on match days (Table 1).

The rate of transmission from cases on match days was 1.55 higher than the rate from cases not infectious on match days as a reference. Similar to the results presented above, the incidence rate ratios (IRRs) based on a negative binomial regression's crude and adjusted rate ratios are provided (see Table 2). Adjusting for sex, the number of daily cases that were infectious, and vaccine status, did not affect the estimate. A sensitivity analysis with the exposure definition ‘infectious on match days’ was replaced by the exposure definition ‘cases that tested positive two days after the match day’ resulted in very similar results, although the effect of match days was reduced to an adjusted IRR of 1.25 (1.15–1.36). Similarly, including an interaction term, combining infectious on match days and the number of days cases were infectious (adjusted IRR 1.06 (1.02–1.10)), reduced the adjusted IRR to 1.16 (1.00–1.34), nor did including 10 year age groups in the model. Model checks, case distribution and sensitivity analysis can be found in the Supplementary materials.

**Table 2.** Incidence rate ratio (IRR) of the number of COVID-19 transmissions in the Danish population from 30 May to 25 July 2021

	Crude			Adjusted^a^		
	IRR	Lower CI (2.5%)	Upper CI (97.5%)	IRR	Lower CI (2.5%)	Upper CI (97.5%)
Infectious at match days *vs*. not infectious at match day	1.55	1.45	1.66	1.41	1.31	1.50
Male sex *vs*. female sex	0.93	0.87	0.98	0.95	0.90	1.01
*N* days from onset of infectiousness to test	1.14	1.12	1.16	1.06	1.04	1.08
One vaccine *vs.* unvaccinated	0.93	0.86	1.02	0.84	0.77	0.92
One vaccine after 14 days *vs.* unvaccinated	0.74	0.68	0.81	0.68	0.62	0.74
Two vaccines *vs.* unvaccinated	0.44	0.36	0.52	0.43	0.36	0.52
Two vaccines after 14 days *vs.* unvaccinated	0.63	0.55	0.72	0.59	0.52	0.68
Symptoms *vs*. no symptoms	1.72	1.61	1.83	1.55	1.45	1.67

a Adjusted for the variables given in the table.

Assuming the crude rate of transmission from each case had been similar for cases not infectious at match days, to cases infectious on match days, using the number of cases as a reference, the expected number of cases not infectious on match days would have resulted in 912 possible transmissions. Using the rate of transmissions by the number of days the cases were infectious, rather than the number of infected cases per infectious day, as the denominator, resulted in 584 possible excess transmissions. The number of transmissions found above represents possible excess transmissions attributable to EC2020 celebrations, assuming similar rates for infectious and non-infectious cases on match days.

### Games at the national arena, Parken and selected big-screen events

For cases, 48 cases were found to be infectious at EC2020 events at the DNSP or big-screen events (of which four were present at more than one event, leaving 44 unique cases). One hundred and twenty-six unique cases were found to be infected after participating in EC2020 events, resulting in 170 unique cases during the events. As attendance to more than one venue occurred, 23 cases were infected after participating in one event, and became infectious at another.

Out of a total of 83 600 spectators present at the four games held at the DNSP in connection with matches on 12, 17, 21 and 28 June 2021, 37 cases were present while infectious, while 117 cases were found positive after participating in the events (see Table 3). Out of 91 565 visitors present in the period from 23 June to 7 July 2021 at big-screen events, 11 cases were found to be present while they were infectious, and 28 persons were found positive after participating in big-screen events. Potentially 126 persons were infected at these events. However, as Table 1 shows, taking WGS data and supporting epidemiological data into account, this number was reduced to 83 possible transmissions to other cases, of which 15 were household transmissions.

**Table 3.** Number of attendants, cases, infected persons, by selected EC2020 events

	NSP, Denmark–Finland 12 June	NSP, Denmark–Belgium 17 June	NSP, Denmark–Russia 21 June	NSP, Croatia–Spain 28 June	Total NSP	Big-screen events	Total
*N* spectators present at event^a^	13 790	23 395	23 644	22 771	83 600	91 565	175 165
*N* infectious at the event^a,b^	1	5	18	13	37	11	48
*N* possibly infected after event^c^	7	42	42	26	117	28	145
*N* unique infected after the event^d^	5	35	35	25	100	26	126
Unique infected within 7 days after the event^d^	4	25	28	20	77	18	95
Incidence	51	180	178	114	140	31	83
7 day incidence by 100 000 persons^e^	29	107	144	92	100	20	54

a Represents total number of attendants some attendants may have attended more than one event and may therefore be counted more than once.

b Persons who were infectious and present at the event, i.e. they tested positive or became symptomatic within 2 days after the event.

c Persons who were not infectious at the event, i.e. persons who were positive, or symptomatic, more than 2 days after an event, but who were associated with the event according to interview data from Danish Patient Safety Authority.

d Persons who were not infectious at the event, i.e. persons who were positive, or symptomatic, more than 2 days after an event, but who were associated with the event according to interview data from Danish Patient Safety Authority, but were only counted as cases if the serial interval indicated that infection was likely.

e The 7-day incidence per 100 000 in the Danish population, using the Danish population of 5 843 347 as reference.

### Genetic distance analysis

The difference in mutations and deletions for transmission between household cases, optimised according to the serial interval, can be seen in Supplementary materials (Fig. S1). Correspondence between the number of deletions and substitutions between primary case–secondary case sequences, the share of household cases and corresponding phylogenetic sequences for the transmissions recorded at the DNSP and big-screen events is visualised and can be seen in Supplementary materials S1 and S2. Although a majority of positive samples were attempted to be sequenced, usable genomes were only extracted from 85% of cases in the cohort (Fig. 1).

## Discussion

In this cohort study, assessing SARS-CoV-2 transmission during EC2020, we saw an increased rate of transmissions to secondary cases, mainly outbreak transmissions, from cases infectious on match days, compared to cases not infectious on match days. Cases infectious on match days also had a higher number of close contacts than those not infectious on match days, and the attack rate was very similar, suggesting the increased number of close contacts was the main driver of the increased number of transmissions. Unadjusted estimates indicated as many as 584 excess cases for persons infectious at match days compared to cases not infectious on match days.

The identified >50% increase in the number of transmissions to secondary cases on match days is higher than findings from other large gatherings, including EC experiences from Scotland, the Sturgis motorcycle rally and other big events [3–5], but less than what can be found in closed events such as bars [19]. Of note, underlying infection rates, restrictions and methodology in examining the effects make any direct comparison difficult. The high focus on contact tracing and that whole sections at DNSP were encouraged to test following attendance might have inflated detection rates, resulting in the high rate of transmissions of 1.8 among cases attending DNSP and big-screen events. Increased identification of persons being infected might have inflated the rate of transmissions and the number of persons identified as infected. Something similar might occur in the general population, with the increased focus on contact tracing and population awareness of symptoms immediately following events. Another explanation could be that persons attending matches might subconsciously suppress symptoms or change test behaviour before the match. Finally, the low attack rate found for cases not attending DNSP or big screen events, the gap between national reproductive number estimates and the number of secondary cases with identified transmission links all reflect the possibility that not all types of transmission were assessed in the current study. If more types of epidemiological ties had been available such as contact tracing data not reaching outbreak threshold, or work relations, the attack rate might have been higher, and the gap to the national effective reproductive number estimate would have been lower in Figure 2. Of note, only cases with a positive RT-PCR test were included in the study. Generally, there was a high level of coherence in the population to the recommendation of a confirmatory RT-PCR test after a positive antigen test in the period up to the EC2020. However, the number of persons not getting a confirmatory RT-PCR test increased during the EC2020, particularly among tourists, potentially leading to an under-detection of cases.

This study has several strengths. First, selection bias was reduced as doctor's appointments and tests are payed for in the universal medical access in Denmark. Further, test capacity is very high, enabling easy access to tests. The Danish registries enable linkage of records linkage, with no loss of follow-up and assessment of vaccination status. Further, the widespread use of WGS enables verification of epidemiologic links as well as and avoidance of spurious links and enables the separation of cases in different lineages. Finally, more than 95% of cases have been in touch with the Danish Patient Safety Authority for the purpose of performing contact tracing. The ability to adjust for several covariates, including vaccination coverage, sex and number of days a case was infectious, is another strength in the study. Even though the two sensitivity analyses, including interaction terms or using another way to assess the effects of match days, pointed towards a lower effect of match days, it is reassuring that the transmission rate to household members was not increased on match days.

The study also harbours some limitations. First, attending games at DNSP, having a case in the household or being connected to an outbreak offers only one of several possible explanations to a SARS-CoV-2 infection. This is also reflected by the low attack rate among contacts found in this study, as several types of possible epidemiological connections, such as transmission, public transport, during work or other social events, are not assessed. The kind of ties hardest to assess are those between persons attending mass events, and in settings without registration, such as those appearing in connection to the EC2020, leading to a possible underestimation of the effects of the EC2020. This limitation applies for all cases in the study period. Second, not all close contacts were registered during contact tracing, as some cases prefer to inform their contacts themselves, and further, only outbreak contacts were assessed. As the above limitation is similar regardless of the date of infection within the period, it is unlikely to affect results between comparator groups, but absolute differences, i.e. the number of cases attributable to the EC2020, are likely to be underestimated. In addition, relaxation of requirements and measures in place might contribute to the rise in cases seen during and after the EC2020. The increased transmissibility of Delta when it was outcompeting the Alpha variant may also have been a contributor to increased transmission. The celebrations taking place at venues such as bars, events in closed environments, with poor ventilation, etc., are also a likely source of transmission, which could arguably be categorised as transmission during the EC2020 but is also not estimated here. Conversely, the vaccination effort increased immunity in the population, reducing the number of susceptible people in the population and test activity among vaccinated individuals. Of note, although a majority of positive samples were attempted to be sequenced, not all resulted in usable genomes.

The spread of COVID-19 is driven by contact between persons. This study found increased transmission among cases infectious at events at the national stadium, though this was mainly due to an increased number of close contacts, an observation also seen in Scotland [5]. The highest incidences found among persons attending the matches at DNSP were higher, than the maximum population incidence found in the timeframe. This could, at least to some extent, be a result of more aggressive contact tracing efforts, or missing data (people not providing information about their attendance at DNSP). However, we did see that the rate of transmissions from SARS-CoV-2 positives that was infectious at the time of the Danish matches, especially outbreak transmissions, was higher than the rate of transmission in the population on days with no matches. We thus find evidence to support the hypothesis that the EC2020 celebrations contributed to the rise in cases during the early summer of 2021 in Denmark, with 584 (1.76%) cases attributable out a total of 33 227 in the period. This number may be even be higher as transmissions from and to tourists attending the games may have gone undetected or might not be detected, resulting in an underestimation of the number of cases attributable to EC2020. Also, cases may in turn infect others or become admitted to the hospital in increased morbidity. Furthermore, not all transmissions were recorded in the current study design. On the other hand, results from the adjusted and sensitivity analysis gave a decreased transmission rate ratio between cases infected at match days and cases infected at other days. Thus the true number of transmissions maybe even smaller, adjusting for the number of days cases were infectious, vaccinations and symptoms. As part of the adjusted regression analysis, a decreased rate of SARS-CoV-2 transmission from fully vaccinated individuals was found and call for further studies with longer timespans, taking age and SARS-CoV-2 lineage into account.

## Conclusion

Our study found that the increased attendance during the EC2020 led to an increase in transmissions. However, professional sports are of great value to many, financially and emotionally. The decision whether to conduct such events during a pandemic should be carefully weighed against possible consequences and the society's ability to withstand the extra cases resulting from increased social activity. Ultimately, combined epidemiological and genetic studies provide valuable tools for assessing and potentially limiting further transmission after such events and help retain normalcy, even in pandemic situations.

## Data Availability

The data in anonymous form are available for access to members of the scientific and medical community for non-commercial use only. Applications should be submitted to Forskerservice at the Danish Health and Medicines Authority, and reviewed based on the relevance and scientific merit. The WGS data that support the findings of this study might be subject to special restrictions.

## Supplementary material

For supplementary material accompanying this paper visit http://doi.org/10.1017/S095026882200019X. click here to view supplementary material
