# Supplementary material for: Increased transmission of SARS-CoV-2 in Denmark during UEFA European championships
Source: Epidemiol Infect. 2022 Mar 23;150:e123. doi: 10.1017/S095026882200019X (PMC9254153; doi:10.1017/S095026882200019X)
Supplement: Supplementary file 1 [file S095026882200019Xsup001.docx]

# Epidemiology and Infection

# Increased transmission of SARS-CoV-2 in Denmark during UEFA European championships.

Marc Bennedbæk , Mia Sarah Fischer Button , Lise Birk Nielsen, Jonas Bybjerg-Grauholm , Christina Wiid Svarrer, Karina Lauenborg Møller , Brian Kristensen, Rebecca Legarth, Vithiagaran Gunalan Ditte Rechter Zenas , Irfatha Irshad, Sophie Gubbels, Raphael N. Sieber , Marc Stegger , Palle Valentiner-Branth, Morten Rasmussen , Camilla Holten Møller, Jannik Fonager , Frederik Trier Moller.

# Supplementary Materials

**Supplementary figure s1**


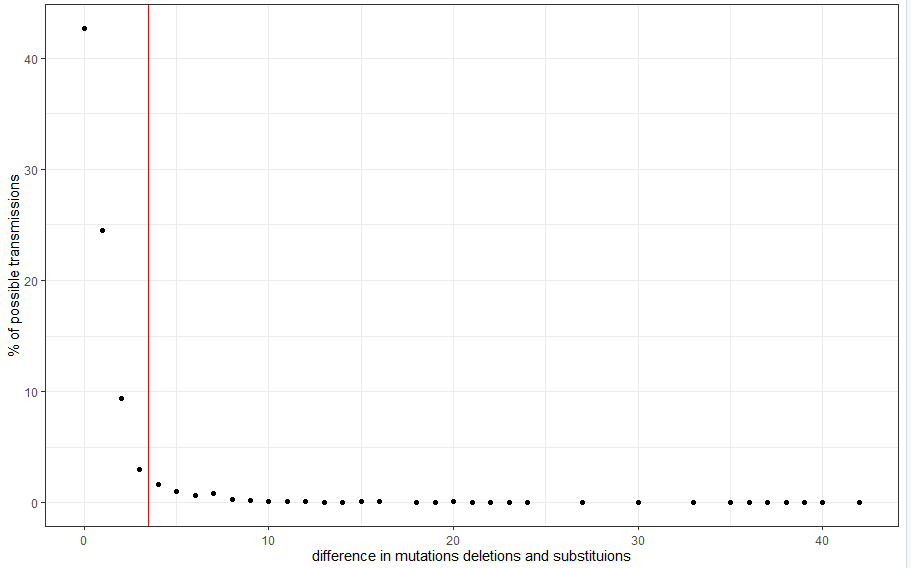


***Figure S one:*** show the difference I mutations deletions and substitutions, for transmission between household cases, optimized according to serial interval, the red line depicts the chosen cut-off

**Supplementary figure S2**


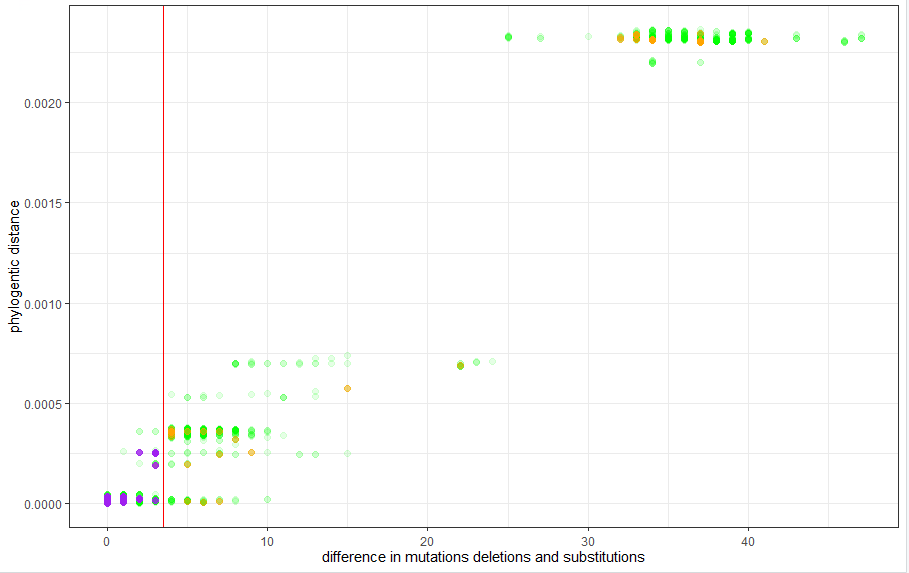


***Figure S two:*** show phylogenetic distances plottet against the difference in number of substitutions and deletions, for cases attending events at the national arena or bigs-screen events. Purple points are the case- case transmission used for analysis, orange points are when the max difference of 3 is not used. Green dots are all possible transmissions.

**Supplementary figure S3**

Panel a


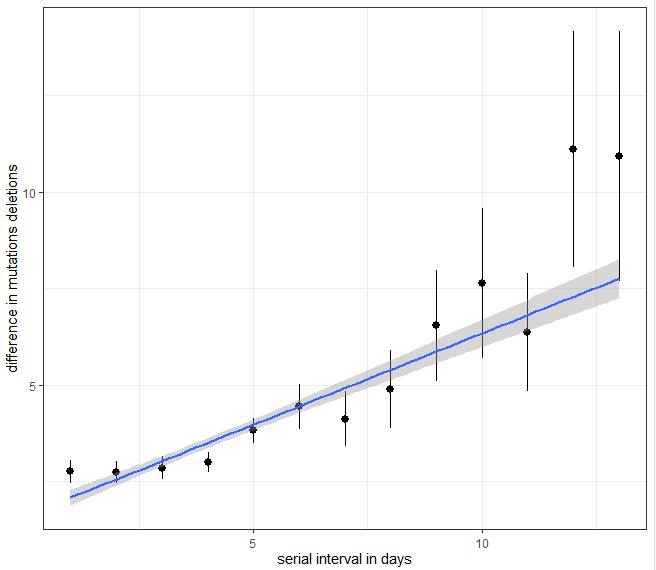


Panel b


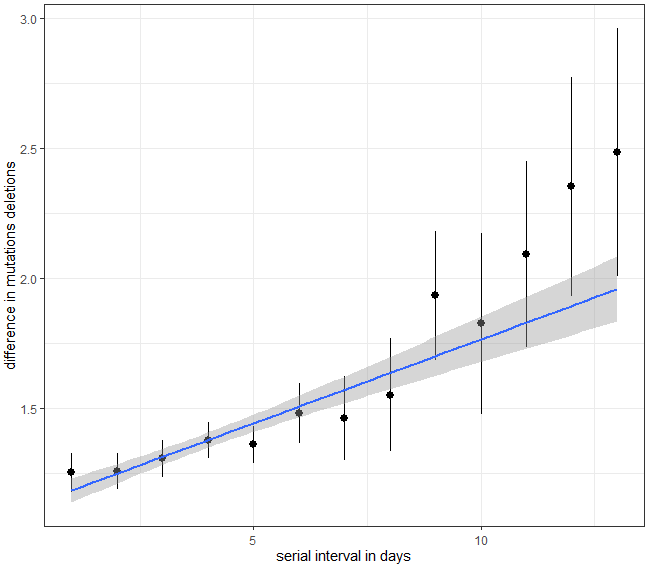


***Figure S 3***: panel a and b shows the difference in mutations and deletions by the serial interval in days between cases before and after pruning the case-case pairs, so that a case can be infected by only one other case.

**Supplementary sensitivity analysis**

**Supplementary table S 1- Incidence rate ratio (IRR) of the number of covid-19 transmissions in the Danish population from** **May 30th to July 25th**

|  | IRR | Lower CI (2.5 %) | Upper CI (97.5 %) | analysis |
| --- | --- | --- | --- | --- |
| Infectious at match days vs not infectious at matchday | 1.55 | 1.45 | 1.66 | crude |
| Male sex vs female sex | 0.93 | 0.87 | 0.98 | crude |
| Number of days infectious | 1.14 | 1.12 | 1.16 | crude |
| One vaccine versus no vaccine | 0.93 | 0.86 | 1.02 | crude |
| One vaccine after 14 days versus no vaccine | 0.74 | 0.68 | 0.81 | crude |
| Two vaccines versus no vaccine | 0.44 | 0.36 | 0.52 | crude |
| Two vaccines after 14 days versus no vaccine | 0.63 | 0.55 | 0.72 | crude |
| Symptoms vs no symptoms | 1.72 | 1.61 | 1.83 | crude |
| Effect of no of infectious days for match days | 1.21 | 1.18 | 1.23 | crude |
| Infectious at match days vs not infectious at match day | 1.16 | 1.00 | 1.34 | adjusted |
| Male sex vs female sex | 0.95 | 0.90 | 1.01 | adjusted |
| Number of days infectious | 1.04 | 1.02 | 1.06 | adjusted |
| One vaccine versus no vaccine | 0.84 | 0.77 | 0.91 | adjusted |
| One vaccine after 14 days versus no vaccine | 0.68 | 0.62 | 0.74 | adjusted |
| Two vaccines versus no vaccine | 0.43 | 0.36 | 0.52 | adjusted |
| Two vaccines after 14 days versus no vaccine | 0.60 | 0.52 | 0.68 | adjusted |
| Symptoms vs no symptoms | 1.56 | 1.45 | 1.67 | adjusted |
| Effect of no of infectious days for non match days | 1.10 | 1.08 | 1.12 | adjusted |
| Interaction with effect of no of infectious days for match days | 1.06 | 1.02 | 1.10 | adjusted |

**Supplementary Table S 2- Incidence rate ratio (IRR) of the number of covid-19 transmissions in the Danish population from** **May 30th to July 25th**

|  | IRR | Lower CI (2.5 %) | Upper CI (97.5 %) | analysis |
| --- | --- | --- | --- | --- |
| Tested positive within 2 days after match days | 1.27 | 1.17 | 1.38 | crude |
| Male sex vs female sex | 0.93 | 0.87 | 0.98 | crude |
| Number of days infectious | 1.14 | 1.12 | 1.16 | crude |
| One vaccine versus no vaccine | 0.93 | 0.86 | 1.02 | crude |
| One vaccine after 14 days versus no vaccine | 0.74 | 0.68 | 0.81 | crude |
| Two vaccines versus no vaccine | 0.44 | 0.36 | 0.52 | crude |
| Two vaccines after 14 days versus no vaccine | 0.63 | 0.55 | 0.72 | crude |
| Symptoms vs no symptoms | 1.72 | 1.61 | 1.83 | crude |
| Tested positive within 2 days after match days | 1.25 | 1.15 | 1.36 | adjusted |
| Male sex vs female sex | 0.96 | 0.90 | 1.01 | adjusted |
| Number of days infectious | 1.07 | 1.05 | 1.09 | adjusted |
| One vaccine versus no vaccine | 0.86 | 0.79 | 0.93 | adjusted |
| One vaccine after 14 days versus no vaccine | 0.69 | 0.63 | 0.75 | adjusted |
| Two vaccines versus no vaccine | 0.43 | 0.35 | 0.51 | adjusted |
| Two vaccines after 14 days versus no vaccine | 0.60 | 0.52 | 0.68 | adjusted |
| Symptoms vs no symptoms | 1,57 | 1,46 | 1,69 | adjusted |

**Supplementary Table S 3- Incidence rate ratio (IRR) of the number of covid-19 transmissions in the Danish population from** **May 30th to July 25^th^ age groups included. All estimates are adjusted.**

| Estimate | IRR | Lower CI (2.5 %) | Upper CI (97.5 %) |
| --- | --- | --- | --- |
| (Intercept) | 0,17 | 0,15 | 0,19 |
| Infectious at match days vs not infectious at matchday | 1,41 | 1,32 | 1,51 |
| Male sex vs female sex | 0,97 | 0,91 | 1,03 |
| Number of days infectious | 1,05 | 1,04 | 1,07 |
| agegrp10-19 | 1,30 | 1,13 | 1,51 |
| agegrp20-29 | 0,76 | 0,66 | 0,88 |
| agegrp30-39 | 1,23 | 1,06 | 1,43 |
| agegrp40-49 | 1,31 | 1,11 | 1,54 |
| agegrp50-59 | 0,97 | 0,80 | 1,17 |
| agegrp60-69 | 0,83 | 0,62 | 1,11 |
| agegrp70-79 | 0,74 | 0,47 | 1,15 |
| agegrp80-89 | 0,84 | 0,41 | 1,65 |
| agegrp90+ | 0,80 | 0,11 | 3,82 |
| One vaccine versus no vaccine | 0,90 | 0,83 | 0,98 |
| One vaccine after 14 days versus no vaccine | 0,71 | 0,65 | 0,78 |
| Two vaccines versus no vaccine | 0,43 | 0,36 | 0,52 |
| Two vaccines after 14 days versus no vaccine | 0,66 | 0,57 | 0,77 |
| Symptoms vs no symptoms | 1,59 | 1,48 | 1,71 |

**Model check main model**


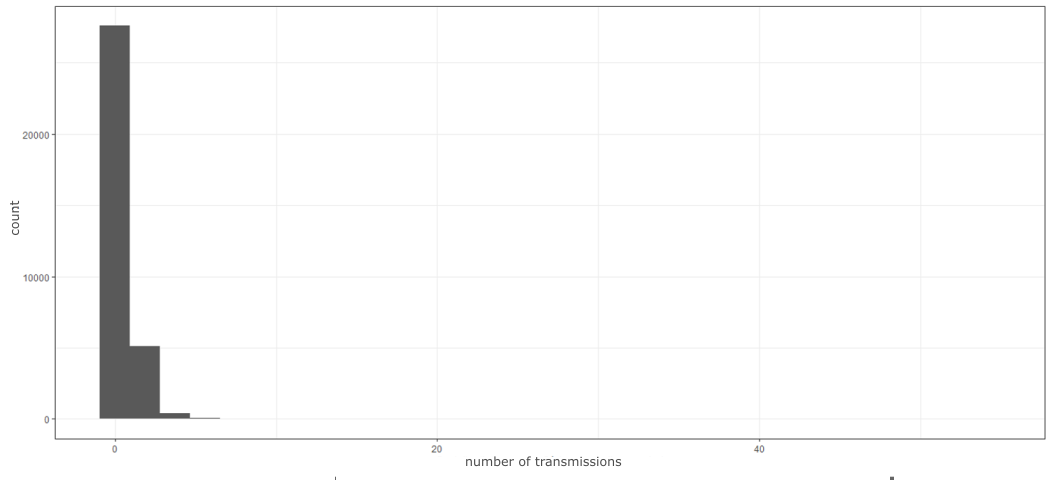


***Figure s 4:*** Shows distribution of number of transmissions from cases

**Supplementary Table S 3 number of possible transmissions identified for each case:**

| tranmissions | 0 | 1 | 2 | 3 | 4 | 5 | 6 | 7 | 8 | 9 | 10 | 11 |
| --- | --- | --- | --- | --- | --- | --- | --- | --- | --- | --- | --- | --- |
| cases | 27574 | 4240 | 881 | 277 | 103 | 48 | 23 | 17 | 10 | 8 | 8 | 5 |
| tranmissions | 12 | 13 | 14 | 15 | 16 | 18 | 19 | 20 | 21 | 23 | 24 | 28 |
| cases | 2 | 4 | 3 | 5 | 2 | 3 | 2 | 1 | 2 | 1 | 1 | 1 |
| tranmissions | 29 | 34 | 54 |  |  |  |  |  |  |  |  |  |
| cases | 1 | 1 | 1 |  |  |  |  |  |  |  |  |  |

The observed variance in the data was 0.8556281

The mean number of contacts was 0.2596382

The predicted number of zero in the model was 25870

The dispersion statistic was 1.488728

**The deviance residuals was:**

Min 1Q Median 3Q Max

-1.0830 -0.6562 -0.5662 -0.4734 8.6787

Null deviance: 16492 on 31262 degrees of freedom

Residual deviance: 15892 on 31254 degrees of freedom

(1964 observations deleted due to missingness)

AIC: 3902

Number of Fisher Scoring iterations: 1

Theta: 0.31531

Std. Err.: 0.00910
